# Bayonet-shaped language development in autism with regression: a retrospective study

**DOI:** 10.1186/s13229-021-00444-8

**Published:** 2021-05-13

**Authors:** David Gagnon, Abderrahim Zeribi, Élise Douard, Valérie Courchesne, Borja Rodríguez-Herreros, Guillaume Huguet, Sébastien Jacquemont, Mor Absa Loum, Laurent Mottron

**Affiliations:** 1 Research Center of the CIUSSS-NIM, Hôpital Rivière-Des-Prairies, 7070, Boul. Perras, Montreal, QC H2E 1A4 Canada; 2 Department of psychiatry, University of Montreal, 2900 Boul. Édouard-Montpetit, Montreal, QC H3T 1J4 Canada; 3 University of Montreal, 2900, Boul. Édouard-Montpetit, Montreal, QC H3T 1J4 Canada; 4 University of Sherbrooke, 2500, Boul. de L’Université, Sherbrooke, QC J1K 2R1 Canada; 5 Sainte-Justine Research Center, 3175, Chemin de La Côte-Sainte-Catherine, Montreal, QC H3T 1C5 Canada; 6 Department of Neurology and Neurosurgery, Montreal Neurological Institute and Hospital, 3801 University Street, Montreal, QC H3A 2B4 Canada; 7 Centre Cantonal Autisme, Centre Hospitalier Universitaire Vaudois and University of Lausanne, Avenue de Beaumont 23, 1011 Lausanne, Switzerland

**Keywords:** Autism, Regression, Speech, Language, Heterogeneity

## Abstract

**Background:**

Language delay is one of the major referral criteria for an autism evaluation. Once an autism spectrum diagnosis is established, the language prognosis is among the main parental concerns. Early language regression (ELR) is observed by 10–50% of parents but its relevance to late language level and socio-communicative ability is uncertain. This study aimed to establish the predictive value of ELR on the progression of language development and socio-communicative outcomes to guide clinicians in addressing parents’ concerns at the time of diagnosis.

**Methods:**

We used socio-communicative, language, and cognitive data of 2,047 autism spectrum participants from the Simons Simplex Collection, aged 4–18 years (mean = 9 years; SD = 3.6). Cox proportional hazard and logistic regression models were used to evaluate the effect of ELR on language milestones and the probability of using complex and flexible language, as defined by the choice of ADOS module at enrollment. Linear models were then used to evaluate the relationship of ELR and non-verbal IQ with socio-communicative and language levels.

**Results:**

ELR is associated with earlier language milestones but delayed attainment of fluent, complex, and flexible language. However, this language outcome can be expected for almost all autistic children without intellectual disability at 18 years of age. It is mostly influenced by non-verbal IQ, not ELR. The language and socio-communicative level of participants with flexible language, as measured by the Vineland and ADOS socio-communicative subscales, was not affected by ELR.

**Limitations:**

This study is based on a relatively coarse measure of ultimate language level and relies on retrospective reporting of early language milestones and ELR. It does not prospectively document the age at which language catches up, the relationship between ELR and other behavioral areas of regression, nor the effects of intervention.

**Conclusions:**

For autistic individuals with ELR and a normal level of non-verbal intelligence, language development follows a “bayonet shape” trajectory: early first words followed by regression, a plateau with limited progress, and then language catch up.

**Supplementary Information:**

The online version contains supplementary material available at 10.1186/s13229-021-00444-8.

## Introduction

One of the first parental concerns leading to an assessment for possible autism is a delay or atypicalities in language and communication [1, 2]. The diagnosis of autism spectrum disorder, most commonly given at a preschool age, occurs frequently when the child may be functionally non-verbal [3]. However, responses to legitimate parental questions related to language outcome, either proximal [4] or even more so as adults [5], are difficult to provide. Apart from the limited proportion of autistic children with typical early language development, the language prognosis of non- or minimally verbal autistic preschoolers is hard to predict [6], even though the proportion of autistic individuals who become fluent by school age is higher than previously thought [7]. The progression of expressive and receptive language development leading to fluency is often not continuous in autism [5, 8] and marked by distinct early phenotypic pathways [9]. The aim of this study was to help clinicians respond to questions about language prognosis at the time of diagnosis of autism spectrum disorder with early language regression (ELR). After apparently normal language and motor development [10], from 10 to 50% of parents of children later diagnosed with autism spectrum disorder note a loss of previously acquired words [11–18]. This loss is associated with a plateau in further language development [10, 16, 19]. The regressive event and stagnation of language progress may be considered either as two aspects of the same phenomenon [19–21] or as two distinct phenomena [22].

Such early language regression (ELR) is rarely encountered in non-autistic children [16, 23, 24]. It occurs at a mean age of 21 months [17], a time when typical developing children experience rapid expansion of their spoken vocabulary [16]. ELR has been suggested to be specific to autism spectrum disorder and is rarely seen in developmental language disorder (DLD) [23]. DLD and autism are considered to be distinct disorders reflecting different etiological mechanisms [25], despite sharing delayed early language milestones and progression, being occasionally comorbid and sometimes difficult to differentially diagnose [23, 26, 27].

Developmental regression was initially associated with poorer language outcomes [14, 28, 29], slow and atypical language development, and unattained complete functional language [13, 29]. Other studies have found that most children with ELR regain their previous language skills [16, 30, 31] after a delay of 4–26 months [11], between 3.5 and 5 years of age [31]. However, it is not clear if ELR affects subsequent language progression or the ultimate level of language attainment.

Large cohort studies support that developmental regression is associated with later higher severity of autism characteristics [32–34] and lower intellectual quotient (IQ) [34, 35]. Both are considered to be predictors of lower language outcome [3, 5, 7]. However, children with ELR generally utter their first words within normal age limits, which is generally not the case for autistic children without ELR [16, 22, 23, 33, 36], and similarly for their first phrase, if ELR occurs after the production of the first phrase [23]. Such early milestones are associated with higher IQ [7] and language level [37–39] in the general autistic population.

Previous studies on regression in the Simons Simplex Collection (SSC) focused on the loss of prelinguistic skills and parental beliefs about the origins of the regression [30, 40]. The progression of language following ELR has been explored little in large cohorts, which focus on the reported duration of the loss and the global level of language, without consideration of language developmental pathways [30, 33]. Studies exploring language outcomes in children with ELR after the age of six years are scarce, with little or no attempt to separate the effect of intellectual disability (ID) from ELR. These studies also did not distinguish between a possible delay in language development and permanent impairment [23, 30, 41, 42].

In this study, we used the SSC to investigate the predictive value of ELR and the effect of NVIQ on language development and late socio-communicative outcomes. A conservative definition of ELR (loss of 5 words for at least three months) was chosen to increase the validity of our retrospective measures [43, 44]. Retrospective information on regression presents a number of limitations: ELR may be less reported by parents of children who quickly regain language [16]. Data on early milestones are also prone to a telescoping effect [16, 45], meaning that the older the child is, the later their milestones are reported. However, caregivers are generally more sensitive to language than other behavioral abnormalities [2, 46] and ELR is the most consistently reported type of regression [44, 47].

Our first objective was to describe the progression of language of autistic children who experienced ELR and to estimate the proportion of them who finally achieved functional and flexible use of language according to their NVIQ. The language level status was provided by the ADOS module used by the clinician at the time of enrollment in the cohort. ADOS module 3 or 4 was chosen if the child had mastered “fluent speech,” i.e., “spontaneous, flexible use of sentences with multiple clauses that describe logical connections within a sentence,” whereas module 2 was used for children possessing only “some flexible phrase” language [48]. The progression of language development was estimated based on the cumulative incidence of early language milestones and the increase in the proportion of fluent speakers, as the age at enrollment in the SSC increased.

Our second objective was to assess the respective effect of ELR on further language and socio-communicative development once fluent speech is reached, while considering NVIQ. As the SSC cohort is cross sectional, we ignored whether non-fluent speakers at enrollment would later develop fluent speech. We thus conservatively focused only on participants with fluent speech at the time of enrollment for this objective. This strategy avoided assuming permanent language impairment based on delayed language development.

## Methods

### Participants

Individuals from the Simons Simplex Collection (SSC) came from 12 university-affiliated research clinics under the guidance of the University of Michigan Autism and Communication Disorders Center. All clinicians received proper training for the administration of the ADOS and ADI-R, with at least 4–6 months of practice, and met standard requirements for research reliability [49]. All individuals included in the SSC were diagnosed with DSM-IV autism, PDD-NOS, or Asperger disorder, based on the clinicians’ best judgment. All participants scored above the threshold on the Autism Diagnostic Observation Schedule (ADOS) and cutoffs in social and communication domains of the Autism Diagnostic Interview-Revised (ADI-R). Participants were between 4 and 18 years of age at enrollment, had no previous additional neurodevelopmental diagnoses, and had a mental age of over 18 months (www.sfari.org). Information from ADI-R on first words, first phrases, and ELR was available for 2,047 autistic participants (mean age = 9.0 years; SD = 3.6). Among this group, 1,707 never experienced ELR (No-ELR), whereas 231 participants experienced ELR after their first words (ELR-W) and 109 after their first phrases (ELR-P) (Table 1). Among them, 1153 individuals (1017 No-ELR, 136 ELR) were fluent speakers at enrollment (based on the use of ADOS module 3 or 4), and complete information on their socio-communicative and cognitive abilities was available. The relative prevalence of epilepsy, which has been suggested to be associated with ELR [50], was not different between the ELR and No-ELR groups (Table 1).

**Table 1:** Socio-demographic data of participants with or without regression

	Fluent and non-fluent speakers				Fluent speakers		
	ELR-W	ELR-P	No-ELR	p-value	ELR	No-ELR	p-value
n (%)	231 (11%)	109 (5%)	1707 (83%)		136 (12%)	1017 (88%)	
Mean age (year: months)	8:9	9:6	9:0	0.23	10:10	10:1	0.011
Gender (male), %	87%	87%	87%	0.98	90%	88%	0.57
*Annual household income, % (n)*							
≤ $ 50_000	21% (45)	20% (20)	15% (250)		14% (18)	15% (148)	
$51,000–$100,000	37% (82)	41% (41)	41% (668)	0.26	44% (56)	43% (417)	0.942
≥ $100,000	42% (92)	40% (40)	44% (719)		42% (54)	42% (412)	
*Mother’s highest level of education, % (n)*							
No college	45% (102)	47% (51)	37% (628)		47% (63)	37% (376)	
College	32% (72)	27% (29)	37% (630)	0.033	30% (40)	37% (372)	0.089
Graduate	23% (53)	27% (29)	26% (445)		24% (32)	26% (267)	
*Mean intellectual quotient (SD)*							
NVIQ	79 (24)	78 (28)	89 (23)	3.3e−13	92 (20)	96 (19)	0.038
NVIQ, pairwise comparisons^a^	79	–	89	3.0e−9			
	–	78	89	1.5e−6			
	79	78	–	1			
VIQ	68 (29)	70 (33)	85 (27)	1.3e−21	88 (22)	95 (21)	4.6e−4
VIQ, pairwise comparisons^a^	68	–	85	1.1e−17			
	–	70	85	2.5e−07			
	68	70	–	1			
VIQ/NVIQ	0.84 (0.23)	0.88 (0.23)	0.95 (0.21)	1.4e−13	0.97 (19)	0.99 (18)	0.081
VIQ/NVIQ, pairwise comparisons^a^	0.84	–	0.95	1.9e−12			
	–	0.88	0.95	2.3e−3			
	0.84	0.88	–	0.40			
EPILEPSY (%)	6 (3%)	4 (4%)	28 (2%)	0.21	3 (2%)	13 (1%)	0.36
Association of epilepsy with ELR, (%)	5 (2%)	4 (4%)			2 (1%)		

For socio-demographic data of non-fluent speakers, see Additional file 3: Table S1

ELR: early language regression, No-ELR: no-early language regression, ELR-W: early language regression after first words, ELRP: early language regression after first phrase, IQ: intellectual quotient, NVIQ: non-verbal IQ, VIQ: verbal IQ

^a^Bonferroni-corrected p-value for pairwise comparisons

### Measures

The ADI-R is a semi-structured, retrospective interview that documents the three behavioral areas relevant for a DSM-IV autism diagnosis [51]. Language regression was determined from ADI-R question #11, as a loss of five or more words for at least three months, the most commonly accepted definition of ELR when using a retrospective questionnaire [17]. Questions #17, #19, #9, and #10 were used to determine the age when the language regression occurred, the duration of the loss, the age of the first meaningful word, and the age of the first two-word phrases including a verb, respectively. The total scores from the Verbal Communication Domain and the Reciprocal Social Interaction Domain of the ADI-R algorithm were used to retrospectively evaluate the historical severity of the socio-communicative impairment. Questions #18 and #85 (in combination with SSC medical history form data) were used to determine whether epileptic attacks were associated with the regressive event according to parental reports and whether the participants had been diagnosed with epilepsy.

The Autism Diagnostic Observation Schedule-Generic (ADOS) is a clinician-administrated semi-structured observational assessment [48]. Modules 1 and 2 of ADOS are used for children who have phrase language at most, but who are not “fluent speakers.” Modules 3 and 4 are used for fluently speaking children or adults. “Fluent speech” is defined as “spontaneous, flexible use of sentences with multiple clauses that describe logical connections within a sentence” [48]. The ADOS calibrated “social affect domain” scores obtained for module 3 [52] and 4 [53] were used to quantify the severity of autism socio-communicative impairment at enrolment of fluent speakers.

The Vineland-Second Edition (VABS) [54] is a standardized, semi-structured, parent or caregiver interview that evaluates adaptive skills for everyday life functioning: communication skills, daily living skills, and socialization. It is one of the most widely used, supported, and validated adaptive behavior scales [55, 56]. It has a high degree of test–retest reliability (internal consistency: 0.72–0.90, inter-rater reliability: 0.78–0.80, test–retest reliability: 0.88–0.92) [54] and excellent test–retest reliability (0.94) in the communication domain in the autistic pediatric population [57]. Two subdomains of the communication domains, receptive and expressive language v-scale scores, were used in this study as measures of expressive and receptive language.

The Peabody Picture Vocabulary 4th edition (PPVT-4) [58] standard score was used as a direct receptive vocabulary measure. In this test, the child has to point to a picture, out of four, corresponding to the word mentioned by the clinician. This measure was used as an additional measure of receptive language focusing on vocabulary knowledge.

The non-word repetition subtest (NWR) standard score from the Comprehensive Test of Phonological Processing [59] is a well-accepted short-term phonological memory task that is impaired in children with DLD [60]. Participants must accurately repeat non-words. This task was used to determine whether any distinction between autistic children with and without ELR could be measured by the NWR task and whether this distinction could contribute to differences in the progression of language development.

Verbal IQ (VIQ) and non-verbal IQ (NVIQ) scores were derived from appropriate psychometric tests. The Differential Ability Scales-Second Edition Early Years/School Age [61], Mullen Scales of Early Learning [62], Wechsler Abbreviated Scale of Intelligence, First Edition [63], and Wechsler Intelligence Scale for Children, 4th Edition [64] were used. For children who failed to complete the age-appropriate test, the IQ was calculated by the formula IQ = (age equivalent score)/chronological age × 100.

### Analyses

The statistical analyses were performed using R software version 3.6.3 [65] (see Additional file 1 for specific packages).

#### Demographics

Chi-square, Kruskal–Wallis, or Wilcoxon signed-rank test analyses were used to compare demographic data. An ANOVA was used to test differences in IQ scores between ELR-W, ELR-P, and No-ELR in the whole sample, followed by group differences with Bonferroni’s correction applied for pairwise comparisons between the ELR-W or ELR-P and No-ELR groups.

#### Language milestones

The time to achieve *first words* and *first phrases* and the time between these milestones are presented as Kaplan–Meier plots for each group (ELR-W, ELR-P, No-ELR). The association between groups and the time at which milestones were achieved were analyzed using Cox proportional hazards models. Hazard ratios (HRs) were obtained for each group. Models were adjusted for NVIQ, sex, and age of assessment to control for any telescoping effect [45]. Logistic-regression analysis was used to estimate the probability of the “fluent speech” status, according to the ADOS module used (fluent speaker: module 3 or 4; not fluent speaker: module 2 or 1), by age, depending on the presence of ELR and the absence of ID (NVIQ ≥ 70) or NVIQ. The interaction between ELR and ID or NVIQ on the probability of being a fluent speaker was also tested. We estimated the elapsed time between the first word/phrase and fluent speech by subtracting the median age of the first word/phrase for each group, from the predicted age at which 50% of each group would be expected to be fluent speakers according to the logistic model.

#### Effects of ELR and NVIQ on socio-communicative and language levels in verbal autistic children

The effect of ELR on socio-communicative and language measures among fluent speakers was quantified using multiple linear regression analyses. Each linear regression was also corrected for NVIQ, sex, and age of assessment for historically reported measures. The socio-communicative and language scores were standardized within the sample for comparability purposes.

## Results

Socio-demographic information on the participants, with or without ELR, for the full sample and those who were fluent speakers at enrollment are presented in Table 1. Sixteen percent of parents reported ELR (11% after the production of first words—ELR-W, and 5% after the first phrases—ELR-P). These proportions remained constant across age at enrollment and were not associated with missing data on ELR, age of first words, or first phrases (Additional file 2: Figure S1), indicating a constant recall bias.

### IQ characteristics of ELR vs No-ELR autistic children

Overall, participants with ELR had a lower IQ than No-ELR participants (NVIQ: *p* = 3.3e−13; ELR-W: 78, ELR-P: 79, No-ELR: 89; VIQ: *p* = 1.3e−21; ELR-W: 68, ELR-P: 70, No-ELR: 85), which was also true when restricted to fluent speakers (NVIQ: *p* = 0.038; ELR: 92; No-ELR: 96; VIQ: *p* = 4.6e−4; ELR: 88, No-ELR: 95). Children with ELR showed a striking discrepancy between the VIQ and NVIQ relative to No-ELR children, as revealed by the VIQ/NVIQ ratio (V/NVIQ ratio: *p* = 1.4e−13; ELR-W: 0.84, ELR-P: 0.88, No-ELR: 0.95). This was mainly driven by non-fluent speakers, as the V/NVIQ ratio did not differ between groups for the fluent speakers (V/NVIQ ratio: *p* = 0.081; ELR: 0.97, No-ELR 0.99) (see Table 1).

### Faster language onset for children with ELR

**Fig. 1:**
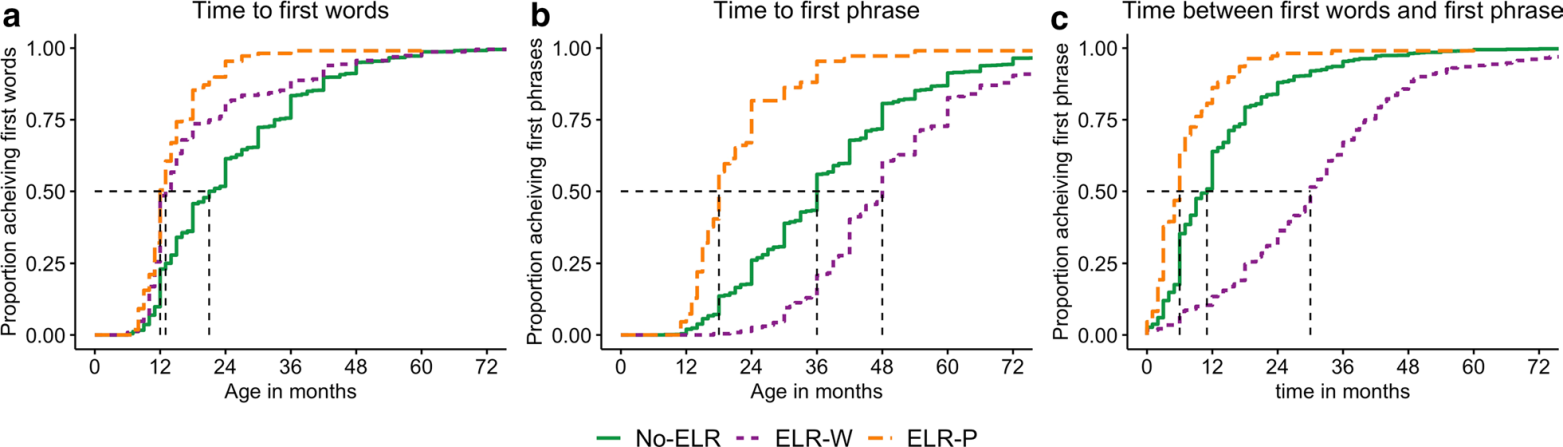
Effect of early language regression (ELR) on language milestones. Proportion of children without language regression (No-ELR), language regression after the production of first words (ELR-W), and language regression after the production of first phrases (ELR-P), achieving language milestones by age/time. **a** Proportion achieving first words by age. **b** Proportion achieving first phrases by age. **c** Proportion achieving first phrase by time, in months, after the first words

First *words* emerged earlier in children who experienced ELR, either before (HR = 1.65, 95% CI [1.44–1.89], *p* = 1.40e−12) or after (HR = 3.32, 95% CI [2.72–4.05], *p* = 2.73e−32) the first phrase. The onset of the first *phrase* also occurred earlier for autistic children with ELR-P (HR = 4.92 95% CI [4.03–6.01], *p* = 9.50e−55), with a shorter time interval between the first words and first phrase than No-ELR children (HR = 2.13, 95% CI [2.07–3.07], *p* = 2.14e−20). As expected, the first phrase was delayed for autistic children with ELR-W (HR = 0.66, 95% CI [0.58–0.76, *p* = 7.04e−09). There was a much longer time interval between first words and the first phrase for ELR-W than No-ELR children (HR = 0.38, 95% CI [0.33–0.44], *p* = 8.92e−41). Overall, ELR occurred in children with earlier initial language onset (see Fig. 1 for descriptive data). Cox models were adjusted for NVIQ, age at enrollment, and sex.

### Non-verbal or minimally verbal plateau following ELR

**Fig. 2:**
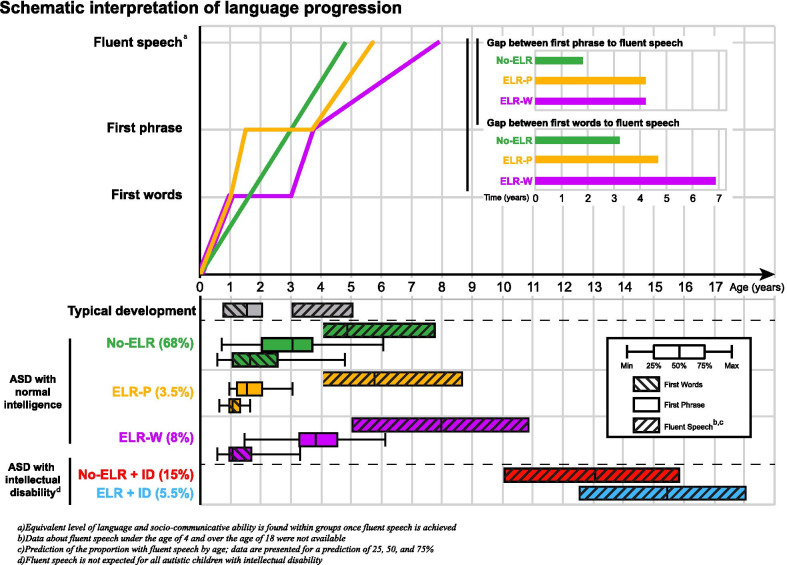
“Bayonet-shaped” language progression of autistic children who experienced early language regression (ELR). Schematic representation of language milestones/age range for autistic children. The progression of language of autistic children with ELR followed a three-step “bayonet-shaped” pattern: early typical language progression followed by a minimally verbal period after ELR and a final catch up phase. The achievement of fluent speech is delayed for children with ELR. Typical development milestones are presented for reference [84]. No-ELR: no-early language regression, ELR-W: early language regression after first words, ELR-P: early language regression after first phrase, ID: intellectual disability (non-verbal IQ < 70)

**Fig. 3:**
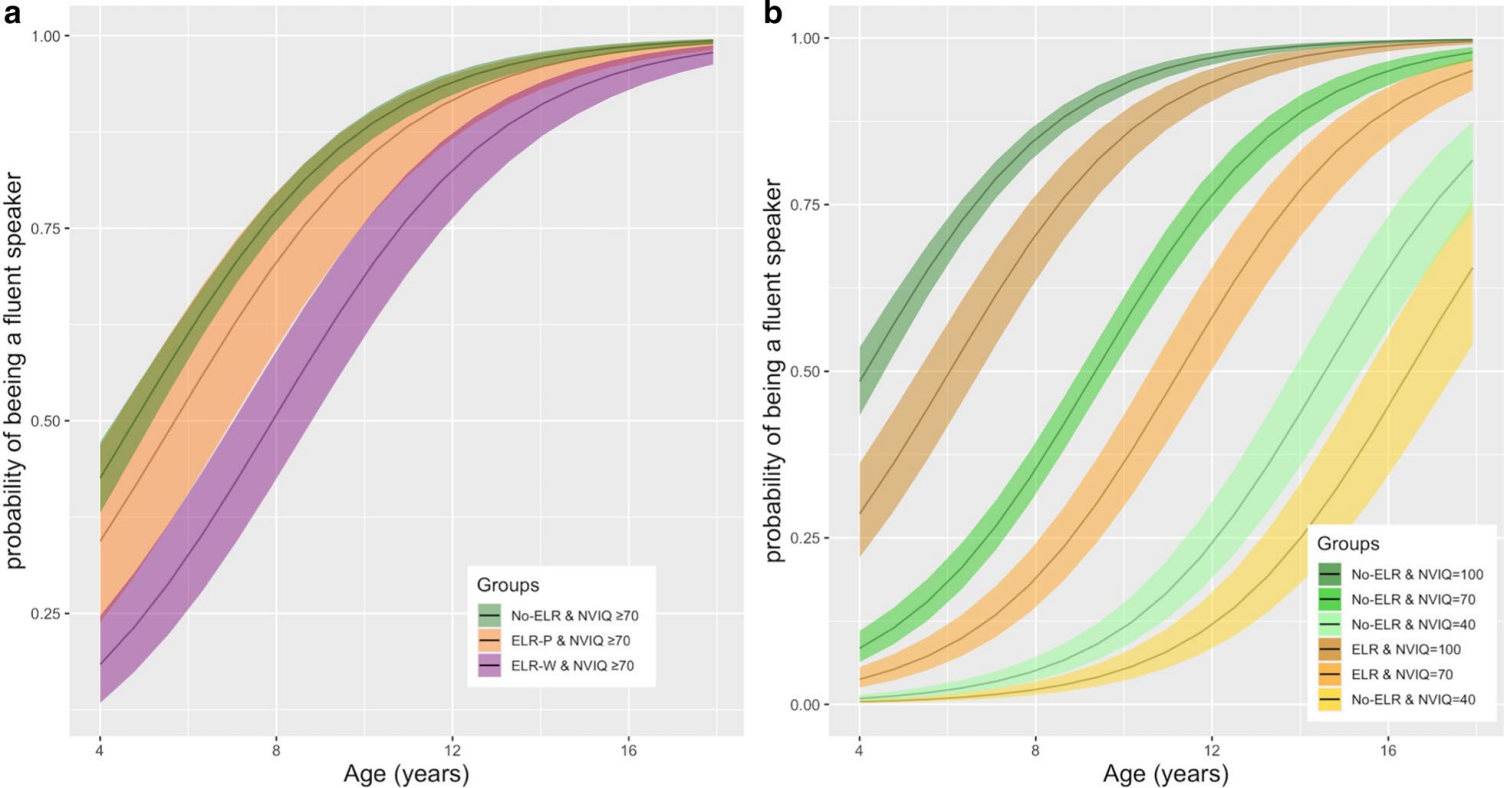
Probabilities of having achieved “fluent speech’’ status by age. Probabilities were derived from a logistic regression, according to a history of early language regression (ELR) and the presence or not of an intellectual disability (ID), with the 95% IC. **a** The probability of being a fluent speaker in non-intellectually disabled participants does not differ between the No-ELR group and that with ELR after their first phrases (ELR-P). Autistic children with a history of ELR after their first words (ELR-W) show delayed development of fluent speech, but still have the same language prognosis at the age of 18. **b** Intelligence explained the fluent speech status considerably more than a history of ELR. Almost all autistic children without intellectual disability (with a non-verbal intellectual quotient ≥ 70) will have achieved fluent speech before the age of 18, whether they have had a history of ELR or not

We evaluated the progression of language development following the first milestones by stratifying for ID, here defined as a NVIQ < 70, which strongly influences further language development [7, 8]. Non-intellectually disabled autistic children who experienced ELR took an average of 21 months to recuperate the language level they had preceding ELR and 50 months between the first phrase and “fluent speech” (Fig. 2). This period was twice the duration observed for autistic children without ELR (ELR-W: 50 months, ELR-P: 50 months, No-ELR: 21 months) (See Figs. 2, 3a). Our study was not sufficiently powered to conduct the same analysis for intellectually disabled children. As expected, ID was strongly associated with a lower probability of being a fluent speaker (OR = 0.045, 95% CI [0.062–0.031], *p* = 1.1e−69). The exclusion of participants with missing information on early language milestones and ELR did not change the magnitude of this effect (Additional file 4: Table S2). ELR also delayed fluent speech (OR = 0.40, 95% CI [0.29–0.53], *p* = 1.16e−9), but to a lesser extent than ID.

### ELR does not lead to a poor language prognosis

More than 97% of non-intellectually disabled autistic children of 18 years of age (ELR: 0.98, 95% CI [0.97–0.99], No-ELR: 0.99 95% CI [0.989–0.996]) are expected to be fluent speakers according to the logistic model, whether they experienced ELR or not. Higher NVIQ was strongly and positively associated with the probability of being a fluent speaker (NVIQ: OR = 1.08, 95% CI [1.07–1.09], *p* = 7.92e−89) (Fig. 3b). The speech status (fluent/not fluent) at the time of enrollment was explained by the NVIQ, rather than a history of ELR (Fig. 3b). There was no interaction effect between ELR and NVIQ or ID on the probability of being a fluent speaker (NVIQ: *p* = 0.19; ID: *p* = 0.30).

**Fig. 4:**
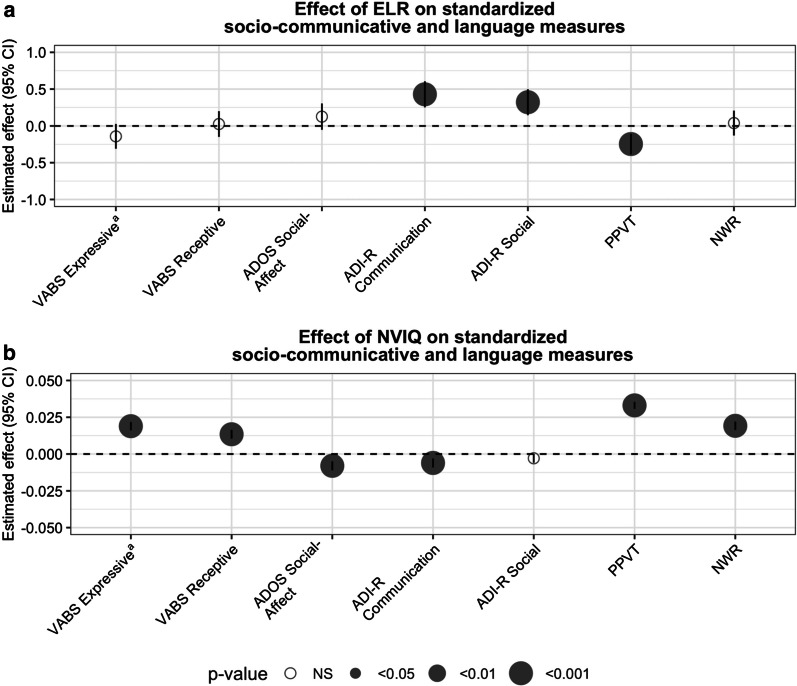
Effects of ELR and non-verbal intelligence quotient on socio-communicative and language measures in fluent speakers. Outcome measures were standardized within the sample to show effect sizes side by side. **a** Effect of early language regression (ELR). ELR did not have a significant effect on the expressive and receptive communicative levels measured by the Vineland (VABS) nor when socio-communicative competence was directly assessed by clinicians (ADOS social affect). Historical measures of impaired communication and social ability, retrospectively reported by parents, are associated with ELR. Lexical knowledge, measured by the PPVT, is negatively affected by ELR. The non-word repetition task score (NWR) is not significantly associated with ELR. Analyses were adjusted for NVIQ, sex, and age of assessment for historical measures. **b** Effect of non-verbal intelligence quotient (NVIQ). NVIQ was positively associated with levels of expressive and receptive language measured by the VABS. Higher NVIQ is protective against the severity of socio-communicative deficit measured by the ADOS. Historical measures of communication are negatively associated with NVIQ, but not social ability when measured retrospectively by the ADIR-R. Lexical knowledge and NWR are both positively associated with NVIQ. Analyses were adjusted for ELR, sex, and age of assessment for historical measures a Effect on the standardized log transformed outcome score

Expressive and receptive language levels of fluent speakers, as reported by parents, were not associated with ELR (z-score VABS_expressive_: ß = −0.14, 95% CI [− 0.31 to 0.026], *p* = 0.099; z-score VABS_receptive_: ß = 0.025, 95% CI [−0.15 to 0.20], *p* = 0.774). This was also true for social-communicative ability, directly assessed by the clinician (z-score ADOS_social affect_: ß = 0.13, 95% CI [−0.052 to 0.30], *p* = 1.6e−1). However, children with ELR were characterized by a history of more socio-communicative impairments than No-ELR children (z-score ADI-R_social_: ß = 0.32, 95% CI [0.14–0.50], *p* = 3.7e−4; z-score ADI-R_communication_: ß = 0.43, 95% CI [0.25–0.61], *p* = 2.2e−6). Receptive vocabulary, measured by the PPVT, but not NWR ability, was lower in fluent speakers with ELR than those with no ELR (z-score PPVT: ß = −0.25, 95% CI [−0.38 to −0.11], *p* = 5.1e−4; z-score NWR: ß = 0.038, 95% CI [−0.13 to 0.21], *p* = 0.65). Analyses were adjusted for NVIQ, sex, and age of assessment for all retrospective measures (see Fig. 4a, Additional file 5: Table S3).

### NVIQ is associated with socio-communicative and language levels

NVIQ was positively associated with expressive and receptive language level of fluent speakers (z-score VABS_expressive_: ß = 0.019, 95% CI [0.016–0.022], *p* = 3.39e−36; z-score VABS_receptive_: 0.013, 95% CI [0.010–0.016], *p* = 2.35e−18) and negatively associated with directly assessed socio-communicative impairment (z-score ADOS_social affect_: ß = −0.0079, 95% CI [−0.011 to −0.0049], *p* = 2.8e−7). NVIQ was negatively associated with past communication impairment (z-score ADI-R_communication_: ß = −0.0060, 95% CI [−0.0091 to −0.0030], *p* = 9.89e−5), but not social impairment (z-score ADI-R_social_: ß = −0.0029, 95% CI [−0.0059 to 0.00016], *p* = 6.29e−2). NVIQ was also positively associated with receptive vocabulary and the NWR score for fluent speakers (z-score PPVT: ß = 0.033, 95% CI [0.031–0.035], *p* < 1e−50; z-score NWR: ß = 0.019 [0.016–0.022], *p* = 5.7e−37). The analyses were adjusted for ELR, sex, and age of assessment for all retrospective measures (see Fig. 4b, Additional file 5: Table S3).

## Discussion

We used cross-sectional data on a large cohort of autistic children of up to 18 years of age to clarify the effect of ELR on language milestones and the probability of becoming a fluent speaker. ELR occurs in children with earlier language onset, as previously shown [23], and delays the achievement of fluent speech. However, it does not affect the probability of their having fluent speech by the age of 18 years, nor undermine the attained expressive and receptive language level measured by the Vineland or socio-communicative ability measured by the ADOS calibrated social affect domain.

Our measurements of language milestones and outcomes were based on parent questionnaires, such as the ADI-R and Vineland, and direct assessment by the PPVT, NWR, and ADOS. Parental reports are prone to recall bias: ELR is suspected to be less reported by parents whose children quickly regain language [16]. Although some have reported the same bias for older children [66], this was not true for our SSC sample. Retrospective measurements, such as the age of milestones or ELR, are also prone to a telescoping effect [16, 45], meaning that the older the child is, the later the milestones are reported. Analyses were corrected for age at assessment to reduce this effect, which in the context of our study was likely a conservative bias. Conversely, knowledge about the diagnosis [67], as well as the parents’ beliefs about the causes [40], could push parents to wrongly report ELR. Despite their suboptimal precision, reports of language regression are the most reliably reported type of regression over time [44] and the most confirmed type of regression when using home-videos [43].

The association of ELR with earlier language onset is mitigated by the fact that only children who have developed language will be able to lose it. However, there was no upper limit for the age at which we considered ELR as such. Hence, one child could have been a “late onset” speaker and still show language regression, but the opposite was found. The presence and timing of regression influenced the duration of the language acquisition plateau, but not the language outcome, extending previous results on the language outcome of ELR [23, 33]. Despite a smaller receptive vocabulary, these children reached the expressive and receptive language level of their peers, according to Vineland parent-reported measures, and to their social communication in a clinical setting (ADOS score). Moreover, ELR was not associated with differences in the NWR task, which usually reveals deficits in DLD [60]. Such a language development pathway and outcome question the interpretation of early language measurements in ELR children as permanent impairment [10]. As suggested previously [37], the slope of language development may be more informative on the language outcome than the level of language observed during the second year of life.

Fluent speakers with ELR presented a more “severe” autistic phenotype in the area of social communication, according to parental recall. This is consistent with the observation that social ability usually regresses parallel to language [31, 68]. It also suggests that the progress in language development in these children may not rely on social ability, which is not required for language development, to the same extent as for typically developing children [69, 70].

A flexible and complex language outcome can be expected for almost all individuals of normal non-verbal intelligence, the achievement of fluent speech being highly associated with NVIQ. This finding is consistent with intelligence being a better indicator than regression to estimate the further needs of autistic children [35]. Conversely, this may also explain why ELR is prospectively associated with greater gains in IQ with age [71], as children who are more verbal become more accessible to intelligence testing. The VIQ was lower than the NVIQ in ELR children in our entire sample. However, the VIQ and NVIQ tended to equalize when evaluated only in fluent speakers, consistent with an initial language onset delay followed by a “catch up” in adolescence [5]. Overall, this emphasizes the importance of assessing non-verbal intelligence, regardless of how challenging this may be in autistic preschool-age children [72]. Although both language and intelligence are related, the two measures are still relatively independent [73, 74].

Language progression before and after ELR follows a three-step, “bayonet-shaped” developmental process: (1) learning first words at an early or typical age [11]; (2) a “plateau” of several years, which doubles the typical time between the first phrase and fluent speech; and (3) catching up to the expressive and receptive language level of their non-regressive autistic peers. Although such a catch-up phase is in agreement with the results of previous studies directly measuring expressive and receptive language in competent talkers [23, 75], it is at odds with those including children over six years of age, which generally showed a lower communicative or language level for autistic children who had had language regression [42, 76]. However, these studies did not consider NVIQ, which significantly influences language development [5].

Prospective declarations concerning the prognosis for language at the time of diagnosis should underline that most non-intellectually disabled autistic children are fluent speakers by the age of eight [7, 8] and ELR does not change their final prognosis. This possibility remains open until at least nine years of age [5]. Finally, our results document the outcome of the plateau in language development that occurs in autistic children when preceded by regression. Regression and plateau may belong to a continuum, the extreme of which is marked by frank regression of language [10, 16, 19–21, 68]. The prevalence of early regression is highly dependent on the definition used [17, 77]. It increases when using more fine-grain questionnaires [30, 31, 78, 79] and reaches 86% when socio-communicative loss/stagnation is used as a criterion in prospective studies, in a population with an elevated likelihood of having ASD [80]. By using a restrictive definition of regression, we classified participants who exhibited subtle forms of regression as non-regressive. ELR retrospectively reported by parents may represent only the most visible fraction of the behavioral or language losses prospectively found in most autistic children [80]. The “bayonet-shaped” language progression curve may represent the more general progression of autistic language development, of which the milder form would be observed in autistic children with subthreshold ELR or a language plateau without evident ELR. This could be verified by further research to ascertain whether the pattern of language development of the two situations is similar or not [11].

Longitudinal studies on children with an elevated likelihood of having ASD [80], such as those using and comparing different measures and definitions of ELR on the progression of language development, are needed to build mechanistic models of language development in autism.

## Limitations

Our study had several limitations. The SSC is composed of participants of simplex families, for whom ELR may be distinct from that of individuals from multiplex families. It is composed of autistic individuals with moderate to severe autistic symptomatology, more representative of autistic children without ID than of those with ID [49]. The study is based on a measure of speech level which does not allow us to detail the difficulties that may persist even in fluent speakers. The predictions made about fluent speech cannot be generalized above and beyond the age range of our sample. We conservatively used a restrictive definition of ELR that relies on parental recall. This may not have identified the entire regressive population, contributing to the low frequency of ELR in this study [17]. The definition of ELR used in this study is representative of the usual information that clinicians have to rely on at the time of diagnosis. There is an inevitable balance between the validity of the measure and the ratio of autistic population concerned by this measure. However, the generalization of our findings to children with ELR identified through other methods should be made with caution. Also, this study did not include control groups with typical children or those with other conditions (e.g., DLD). This limits our ability to contextualize the language developmental profile of autistics with ELR to other "late bloomers" seen in other conditions [81]. Given the cross-sectional nature of this study, NVIQ levels were those measured at the time of enrollment. Thus, this study did not consider the evolution of NVIQ over time. The NVIQ measured at enrollment is at risk of being underestimated in younger children without ID [82, 83]. The NVIQ may also have been affected by numerous factors, such as ELR, which tends to be associated with an underestimation of IQ at a young age [71]. Retrospective data on socio-communicative impairment, extracted from the ADI-R, document the most intense level of abnormalities presented or at the age at which abnormalities were the most obvious. Such measures are thus subject to temporal imprecision. Finally, our study does not provide information on the relationship between language regression and other behavioral areas of regression, nor on atypical pre-regression “early-onset’’ features. It also does not document the effects of intervention or their absence in the developmental course of language.

## Conclusion

Responses to parents’ concerns relating to language outcome should highlight the fact that ELR does not affect the late language prognosis, but that it may delay its progression. The language development associated with ELR follows a ''bayonet” shape: early first words, regression, plateau, and language catch-up. Regardless of the etiological relationship between intelligence and ELR, NVIQ is strongly associated with fluent speech. Without NVIQ assessment, ELR should not be used as a predictor for a poor language prognosis, and a strong autistic phenotype in the socio-communicative domain at a young age does not necessarily overlap with a poorer language prognosis. Characterizing the progress, as well as the quality of the language “plateau,” with prospective studies may lead to a better understanding of language acquisition in autism. Finally, the “catching up” of language abilities after a plateau, if intrinsic to the development of language in autism, represents a confounding variable to be considered in any measurement of the effect of intervention on later language level.

## Supplementary Information

**Additional file 1**. List of R packages used for the analyses.

**Additional file 2**. Figure S1. Prevalence and relative prevalence of early language regression (ELR) in the original sample.

**Additional file 3**. Table S1. Socio-demographic data of non-fluent participants with or without ELR.

**Additional file 4**. Table S2. Effect of intellectual disability and age on the prediction of being a fluent speaker.

**Additional file 5**. Table S3. Effects of ELR, NVIQ, age, and sex on standardized socio-communicative and language levels in fluent speakers.

## Data Availability

The dataset used for the current study is available for approved researchers by applying at https://base.sfari.org.

## Abbreviations

ADI-R: Autism diagnostic interview-revised
ADOS: Autism diagnostic observation schedule
DLD: Developmental language disorder
ELR: Early language regression
ELR-W: Early language regression after first words
ELR-P: Early language regression after first phrases
ID: Intellectual disability
IQ: Intelligence quotient
NVIQ: Non-verbal intelligence quotient
NWR: Non-word repetition task
PPVT: Peabody picture vocabulary test
SSC: Simons simplex collection
VABS: Vineland adaptive behavior scales
VIQ: Verbal intelligence quotient
