# Supplementary material for: Bayonet-shaped language development in autism with regression: a retrospective study
Source: Mol Autism. 2021 May 13;12:35. doi: 10.1186/s13229-021-00444-8 (PMC8117564; doi:10.1186/s13229-021-00444-8)
Supplement: Supplementary file 1 — Additional file 1. List of R packages used for the analyses. [file 13229_2021_444_MOESM1_ESM.docx]

**Supplement 1**

*List of R packages used for the analyses*

For built-in library[1]

- Logistic regressions: glm( )
- Linear regressions: lm()
- ANOVA: aov()
- Kruskal-Wallis rank sum test: kruskal.test()
- Wilcoxon rank test: wilcox.test()
- Chi-squared test: chisq.test()

For specific packages:

- Cox proportionate hazards models:
  - R package “survival”[2,3]; coxph()

Supplementary references:

[1] R Core Team. *R: A Language and Environment for Statistical Computing*. Vienna, Austria: R Foundation for Statistical Computing, https://www.R-project.org (2020).

[2] Therneau TM. *A Package for Survival Analysis in S*, https://CRAN.R-project.org/package=survival (2015).

[3] Therneau TM, Grambsch PM. *Modeling survival data: extending the Cox model*. New York: Springer, 2000.
