# Supplementary material for: Bayonet-shaped language development in autism with regression: a retrospective study
Source: Mol Autism. 2021 May 13;12:35. doi: 10.1186/s13229-021-00444-8 (PMC8117564; doi:10.1186/s13229-021-00444-8)
Supplement: Supplementary file 2 — Additional file 2. Figure S1. Prevalence and relative prevalence of early language regression (ELR) in the original sample. [file 13229_2021_444_MOESM2_ESM.docx]

**
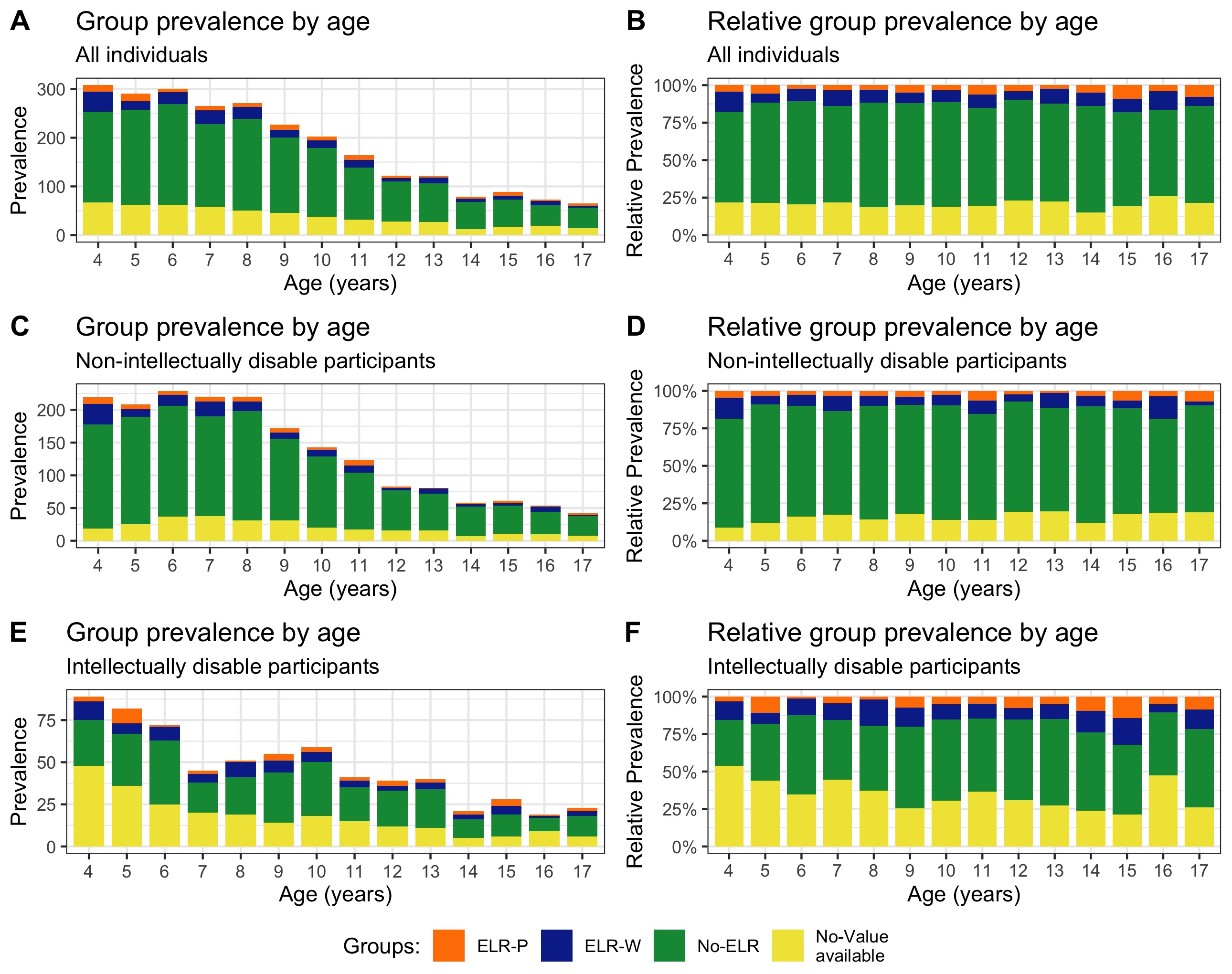
**

| **Figure S1. Prevalence and relative prevalence of early language regression (ELR) in the original sample**. *Participants without language regression (No-ELR), with language regression after the production of first words (ELR-W), and language regression after the production of first phrases (ELR-P). Participants excluded due to lack of information are shown in yellow.* ***(A, B)*** *Without stratification for the non-verbal intellectual quotient (NVIQ), there was no difference in the relative prevalence of the groups by age, p = 0.45.* ***(C, D)*** *For the non-intellectually disabled participants (NVIQ ≥ 70), there was no difference in the relative prevalence of the groups by age, p = 0.11.* ***(E, F)*** *For the intellectually disabled participants (NVIQ < 70), there was no difference in the relative prevalence of the groups by age, p = 0.18.* |
| --- |
