# Supplementary material for: Bayonet-shaped language development in autism with regression: a retrospective study
Source: Mol Autism. 2021 May 13;12:35. doi: 10.1186/s13229-021-00444-8 (PMC8117564; doi:10.1186/s13229-021-00444-8)
Supplement: Supplementary file 3 — Additional file 3. Table S1. Socio-demographic data of non-fluent participants with or without ELR. [file 13229_2021_444_MOESM3_ESM.docx]

**Table S1.** Socio-demographic data of non-fluent participants with or without ELR

|  | Non-Fluent speakers | | |  |
| --- | --- | --- | --- | --- |
|  | ELR | No-ELR | p-value | |
| n (%) | 187 (%) | 551 (%) |  | |
| mean age (year:months) | 7:11 | 7:3 |  | |
| gender (male), % | 84% | 83% | 0.88 | |
|  |  |  |  | |
| ANNUAL HOUSEHOLD INCOME, % (n) |  |  |  | |
| ≤ $ 50_000 | % (43) | % (90) | 0.088 | |
| $ 51 000 to $ 100 000 | % (62) | % (192) |  |  |
| ≥ $ 100 000 | % (71) | % (244) |  |  |
|  |  |  |  | |
| MOTHER’S HIGHEST LEVEL OF EDUCATION, % (n) |  |  |  | |
| no college | 46% (84) | 37% (203) | 0.068 | |
| college | 29% (54) | 38% (207) |  |  |
| graduate | 25% (46) | 25% (140) |  |  |
|  |  |  |  | |
| MEAN INTELLECTUAL QUOTIENT (SD) |  |  |  | |
| NVIQ | 68 (24) | 74 (23) | 0.003 | |
| VIQ | 52 (25) | 63 (25) | 3.3e-7 | |
| VIQ/NVIQ | 0.76 (0.21) | 0.85 (0.24) | 2.1e-6 | |
|  |  |  |  | |
| EPILEPSY (%) | 7 (4%) | 15 (3%) |  | |
| ASSOCIATION OF EPILEPSY WITH ELR,(%) | 7 (4%) |  |  | |
| **Note**: ELR: early language regression, No-ELR: no-early language regression, IQ: intellectual quotient, NVIQ: non-verbal IQ, VIQ: verbal IQ  *Non-fluent speakers at time of enrollment are composed of two indistinguishable subgroups; those who will remain nonverbal, and those who will become fluent speakers after enrollment. | | | |  |
