# Supplementary material for: Bayonet-shaped language development in autism with regression: a retrospective study
Source: Mol Autism. 2021 May 13;12:35. doi: 10.1186/s13229-021-00444-8 (PMC8117564; doi:10.1186/s13229-021-00444-8)
Supplement: Supplementary file 4 — Additional file 4. Table S2. Effect of intellectual disability and age on the prediction of being a fluent speaker. [file 13229_2021_444_MOESM4_ESM.docx]

**Table S2.** Effects of intellectual disability and age on the prediction of being a “fluent speaker”

|  |  | **NVIQ ≥ 70** | | **Age (months)** | |
| --- | --- | --- | --- | --- | --- |
|  | **n** | **OR**  **[95% CI]** | **p-value** | **OR**  **[95% CI]** | **p-value** |
| **Sample used in the study** | 2047 | 23.72  [16.96 to 33.74] | < 1e-50 | 1.46  [1.40 to 1.52] | < 1e-50 |
| **Original sample with excluded participants** | 2577 | 34.88  [25.72 to 48.03] | < 1e-50 | 1.44  [1.39 to 1.50] | < 1e-50 |

**Note**: *Effects are shown for the original sample and the sub-sample used in this study. The effect of early language regression was excluded from these analyses, as the excluded participants did not have this information. NVIQ: non-verbal intellectual quotient*
