# Supplementary material for: Bayonet-shaped language development in autism with regression: a retrospective study
Source: Mol Autism. 2021 May 13;12:35. doi: 10.1186/s13229-021-00444-8 (PMC8117564; doi:10.1186/s13229-021-00444-8)
Supplement: Supplementary file 5 — Additional file 5. Table S3. Effects of ELR, NVIQ, age, and sex on standardized socio-communicative and language levels in fluent speakers. [file 13229_2021_444_MOESM5_ESM.docx]

**Table S3.** Effects of ELR, NVIQ, age, and sex on standardized socio-communicative and language levels in fluent speakers

|  | ***ELR*** | | ***NVIQ*** | | ***Age at assessment***  ***(months)*** | | ***Sex*** | |
| --- | --- | --- | --- | --- | --- | --- | --- | --- |
|  | **ß**  **[I.C. 95%]** | **p-value** | **ß**  **[I.C. 95%]** | **p-value** | **ß**  **[I.C. 95%]** | **p-value** | **ß**  **[I.C. 95%]** | **p-value** |
| **Socio-communicative** |  |  |  |  |  |  |  |  |
| Vineland Expressive ^a^ | -0.14  [-0.31 to 0.026] | 9.9e-02 | 0.019  [0.016 to 0.022] | 3.4e-36 | - | - | -0.042  [-0.21 to 0.12] | 6.2e-01 |
| Vineland Receptive | 0.025  [-0.15 to 0.20] | 7.7e-01 | 0.013  [0.010 to 0.016] | 2.4e-18 | - | - | -0.077  [-0.25 to 0.095] | 3.8e-01 |
| ADOS Social affect | 0.13  [-0.052 to 0.30] | 1.6e-01 | -0.0079  [-0.011 to -0.0049] | 2.8e-7 | - | - | 0.062  [-0.11to 0.24] | 4.9e-01 |
| ADI-R verbal communication total score | 0.43  [0.25 to0.61] | 2.2e-06 | -0.0060  [-0.0091 to -0.0030] | 9.9e-05 | 0.00078  [-0.00068 to 0.0022] | 2.9e-01 | 0.030  [-0.15 to 0.21] | 7.4e-01 |
| ADI-R Social total score | 0.32  [0.14 to 0.50] | 3.7e-04 | -0.0029  [-0.0059 to 0.00016] | 6.3e-02 | 0.0034  [0.0019 to 0.0049] | 5.1e-06 | 0.18  [0.0085 to 0.36] | 4.0e-02 |
| **Language** |  |  |  |  |  |  |  |  |
| PPVT standard score | -0.25  [-0.38 to -0.11] | 5.1e-04 | 0.033  [0.031 to 0.035] | <1e-50 | - | - | -0.14  [-0.28 to -0.0057] | 4.1e-02 |
| NWR standard score | 0.038  [-0.13 to 0.21] | 6.5e-01 | 0.019  [0.016 to 0.022] | 5.7e-37 | - | - | -0.10  [-0.27 to 0.062] | 2.2e-01 |

**Note***: ELR: early language regression, NVIQ: non-verbal intellectual quotient*

*^a^coefficient affecting the standardized log transformed outcome score*
